# Phase II study of olaparib in patients with refractory Ewing sarcoma following failure of standard chemotherapy

**DOI:** 10.1186/1471-2407-14-813

**Published:** 2014-11-05

**Authors:** Edwin Choy, James E Butrynski, David C Harmon, Jeffrey A Morgan, Suzanne George, Andrew J Wagner, David D’Adamo, Gregory M Cote, Yael Flamand, Cyril H Benes, Daniel A Haber, Jose M Baselga, George D Demetri

**Affiliations:** Division of Hematology Oncology, Massachusetts General Hospital, Boston, MA USA; Medical Oncology, Dana Farber Cancer Institute, Boston, MA USA; Department of Statistics, Dana Farber Cancer Institute, Boston, MA USA; Massachusetts General Hospital Cancer Center, Boston, MA USA; Ludwig Center at Dana-Farber/Harvard Cancer Center, Boston, MA USA; Memorial Sloan Kettering Cancer Center, New York, NY USA; 55 Fruit St, Boston, MA 02114 USA

## Abstract

**Background:**

Preclinical studies have documented antitumor activity of PARP inhibition both *in vitro* and *in vivo*, against Ewing sarcoma cells. This study aimed to translate that observation into a clinical trial to assess the efficacy and tolerability of olaparib, a PARP inhibitor, in patients with advanced Ewing sarcoma (EWS) progressing after prior chemotherapy.

**Methods:**

In this nonrandomized phase II trial, adult participants with radiographically measureable metastatic EWS received olaparib tablets, 400 mg orally twice daily, until disease progression or drug intolerance. Tumor measurements were determined by CT or MRI at 6 and 12 weeks after starting olaparib administration, and then every 8 weeks thereafter. Tumor response determinations were made according to RECIST 1.1, and adverse event determinations were made according to CTCAE, version 4.0. A total of 22 participants were planned to be enrolled using a conventional 2-step phase II study design. If no objective responses were observed after 12 participants had been followed for at least 3 months, further accrual would be stopped.

**Results:**

12 participants were enrolled, and all were evaluable. There were no objective responses (PR/CR), 4 SD (duration 10.9, 11.4, 11.9, and 17.9 wks), and 8 PD as best response. Of the SD, 2 had minor responses (−9% and −11.7% by RECIST 1.1). The median time to disease progression was 5.7 weeks. Further enrollment was therefore discontinued. No significant or unexpected toxicities were observed with olaparib, with only a single case each of grade 3 anemia and grade 3 thrombocytopenia observed.

**Conclusions:**

This study is the first report of a prospective phase II trial to evaluate the safety and efficacy of a PARP inhibitor in patients with advanced Ewing sarcoma after failure of standard chemotherapy. Olaparib administration was safe and well tolerated when administered to this small heavily pre-treated cohort at the 400 mg BID dose, although the median duration of dosing was for only 5.7 weeks. No significant responses or durable disease control was seen, and the short average interval to disease progression underscores the aggressiveness of this disease. Other studies to combine cytotoxic chemotherapy with PARP inhibition in EWS are actively ongoing.

**Trial registration:**

ClinicalTrials.gov Identifier: NCT01583543

## Background

Ewing sarcoma is a highly malignant tumor of either bone or soft tissue that occurs most frequently in the adolescent and young adult years [1]. The cell of origin of Ewing sarcoma remains poorly defined, however a neuroectodermal origin is suspected. Although a rare disease, it is the 2nd most common primary bone tumor of childhood. With the advent of adjuvant chemotherapy, the prognosis of localized Ewing sarcoma has improved from less than 20% to currently greater than 70% survival in 5-years. However, patients with recurrent Ewing sarcoma have poor prognosis [2–5]. Few patients with recurrences become long-term survivors, and they are usually limited to those with local recurrence and a long initial remission [4, 6, 7]. Second line and subsequent chemotherapy has had limited success and is associated with significant toxicity. There is no single accepted standard of care that is highly effective for these patients, and complete responses are rare. Five-year event-free and overall survival following recurrence is less than 15% [3, 5, 8]. The prognosis is even worse for patients who relapse within two years after diagnosis and patients who have distant recurrences that are not treatable with radical surgery. In a single institution longitudinal experience over 20 years, 215 patients of an initial cohort of 402 patients (53.5%) relapsed. Of these relapsed patients, 200 (93%) died with a mean survival of 13.1 months and no patient with extrapulmonary metastases survived [8].

Garnett et al. observed that Ewing sarcoma cell lines were over 100-fold more sensitive to PARP inhibition with olaparib (AZD2281, KU-0059436), a potent Polyadenosine 5′diphophoribose (poly ADP ribose) polymerase (PARP) inhibitor, than were control cell lines, and treatment with Olaparib selectively induced apoptosis [9]. This level of sensitivity was comparable to that observed in BRCA2 deficient cells. Olaparib has been shown to inhibit selected tumor cell lines in vitro and in xenograft and primary explant models as well as in genetic BRCA knock-out models, either as a stand- alone treatment or in combination with established chemotherapies [10–12]. Brenner et al. additionally showed that PARP inhibition by olaparib potentiated DNA damage induced by expression of EWS-FLI1 or EWS-ERG fusion genes, thereby inhibiting growth of tumor subcutaneously implanted into SCID mice [13]. Based on these results, we performed a single arm open labeled clinical trial, constructed along a conventional Simon 2-step phase II study design [14], to evaluate the safety and clinical activity of olaparib in adult patients with advanced Ewing sarcoma following failure of conventional chemotherapy. (ClinicalTrials.gov Identifier: NCT01583543).

## Methods

This study is a single arm, open label, phase II study to investigate the clinical efficacy and safety of olaparib in patients with metastatic and/or recurrent Ewing sarcoma. Pathologic diagnosis of Ewing sarcoma had to be confirmed by pathologic review at one of the participating institutions, but molecular testing for an EWS translocation was not required for eligibility.

A single consortium, the Dana-Farber/Harvard Cancer Center completed the study through two active sites (Dana-Farber Cancer Institute and Massachusetts General Hospital).

The study was designed to distinguish a favorable true response rate of 24% from a null rate of 5% using a conventional 2-step phase II study design model [14]. The first group of 12 subjects was to be enrolled. If no patient experienced response after all 12 subjects had been followed for at least 3 months, further accrual would be stopped and the study drug would be declared as ineffective. If 1 or more patients experienced response, an additional 10 patients would be enrolled to a total study population of N = 22. If 3 or more patients among 22 eligible, treated patients experience response, the drug would be considered effective and worthy of further study. Study-wide response rates were to be estimated after all subjects had been followed for at least 6 months. This statistical model assumes a null versus alternative response rate as 5% versus 24%, with a 9% type I error and 91% power. This design had a 54% probability of stopping early if the drug was ineffective.

Olaparib was administered orally at 400 mg (tablet formulation) twice daily. Participants were to take Olaparib twice daily, without break, and were supplied a sufficient quantity on Day 1 of the study to last until the second study visit (Day 43). After Day 1 of the study additional visits occurred after 6 weeks, 12 weeks, and then every 8 weeks thereafter. Subjects were instructed not to make up for vomited doses, and to record missed or vomited doses on the Patient Diary Card. Safety and tolerability were monitored continuously throughout study participation.

Tumor assessments using CT scans of the chest, abdomen, and pelvis were done at baseline and then repeated after 6 weeks, 12 weeks, and then every 8 weeks thereafter to assess disease status.

In the absence of treatment delays due to adverse events, study drug administration was to be continued until one of the following criteria applied: Disease progression by RECIST 1.1, intercurrent illness that prevented further administration of treatment, unacceptable adverse events, decision of participant to withdraw from study, general or specific changes in the participant’s condition that rendered the participant unacceptable for further treatment in the opinion of the treating investigator, or bone marrow findings consistent with myelodysplastic syndrome/acute myeloid leukemia.

The study was approved by the Dana-Farber/Harvard Cancer Center Institutional Review Board, and all patients signed informed consent prior to study registration. This study was opened to accrual on May 25, 2012, and closed to accrual on February 25, 2013. All patients were to be followed for at least 30 days after removal from study or until death. Participants who were removed from study for unacceptable adverse events were to be followed until resolution or stabilization of the adverse event.

## Results

Table 1 displays patient demographics and other characteristics at baseline. The median age was 25.5 years (range 18 to 70 years). 100% of subjects were white, and 10 (83%) were male, while 2 (17%) were female. 9 subjects (75%) had prior surgical treatment, and 9 subjects (75%) had prior radiation treatment. The median number of prior radiation treatments was 1 and the median number of prior chemotherapy treatments was 5.

**Table 1:** **Baseline characteristics**

Age (in years, at date of registration)	
Mean	30.5
Std	15.38
Min	18
Max	70
Gender	
Male	N=10, 83%
Female	N=2, 17%
Institution	
DFCI	N=4, 33.3%
MGH	N=8, 66.7%
Performance status	
0	N=6, 50%
1	N=6, 50%
Prior surgery	
Yes	N=9, 75%
No	N=3, 25%
Prior radiation	
Yes	N=9, 75%
No	N=3, 25%
Number of prior radiation treatments	
N (patients)	9
Mean (#prior treatments)	1.7
Std	1.1
Median	1.0
Min	1
Max	4
Prior Chemotherapy	
Yes	N=12, 100%
Number of prior chemotherapy treatments	
N (patients)	12
Mean (#prior treatments)	6.5
Std	6.4
Median	5
Min	1
Max	20

Table 2 displays a summary of treatment-related adverse events (counts by worst grade per patient, and worst grade per patient across all toxicities). No deaths were experienced on this study. There was one occurrence of a Grade 4 event (patient was hospitalized with a pneumonia), and 6 occurrences of Grade 3 events (pain, anemia, lymphocyte count decreased and platelet count decreased) across all toxicities. The grade 4 event and 2 of the grade 3 events (pain) were not attributed to the study drugs or procedures by the study investigators. No patients were taken off study due to an unacceptable adverse event. One patient did not experience any toxicity while on study treatment.

**Table 2:** **Toxicity maximum grade per patient – attributable to treatment**

Toxicity type	Description	Grade		
		**1**	**2**	**3**
BL101	Anemia	0	0	2
BL999	Blood and lymphatic system disorders - Other	1	0	0
CN108	Fatigue	3	0	0
CN109	Fever	2	0	0
CN121	Non-cardiac chest pain	1	0	0
GI121	Constipation	1	0	0
GI123	Diarrhea	1	0	0
GI124	Dry mouth	2	0	0
GI179	Nausea	2	1	0
GI210	Stomach pain	1	0	0
GI216	Vomiting	3	0	0
GI999	Gastrointestinal disorders - Other	1	0	0
IN171	Urinary tract infection	1	0	0
IV127	Lymphocyte count decreased	0	0	1
IV131	Platelet count decreased	0	1	1
ME999	Metabolism and nutrition disorders - Other	2	0	0
MU112	Generalized muscle weakness	1	0	0
MU999	Musculoskeletal and connective tissue disorder - Other	1	0	0
NE118	Dysgeusia	1	0	0
NE126	Headache	1	0	0
PU113	Cough	1	0	0
PU999	Respiratory, thoracic and mediastinal disorders - Other	1	0	0
VA102	Flushing	1	0	0
VA104	Hot flashes	1	0	0
**TOTAL**		**29**	**2**	**4**
**Worst grade per patient across all types**				
			**Count**	**Percentage**
**Mild - grade 1 as worst degree**			5	45.45%
**Moderate - grade 2 as worst degree**			1	9.09%
**Severe - grade 3 as worst degree**			4	36.36%
**Life threatening**			1	9.09%

**Figure 1:**
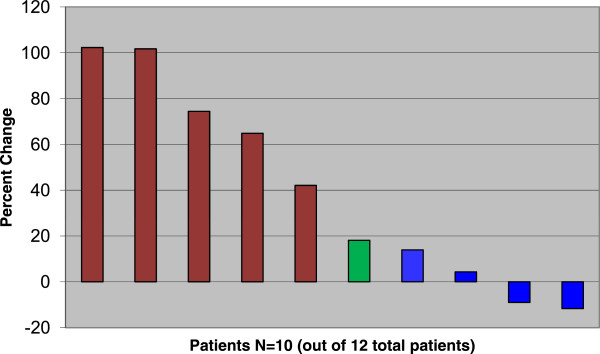
**Percent change from baseline in sum of longest diameter of target lesions.** Red: Progressive Disease, Green: Progressive Disease due to development of new metastatic lesions, Blue: Stable Disease. Two patients did not receive post-treatment imaging due to rapid clinical progression of disease.

**Figure 2:**
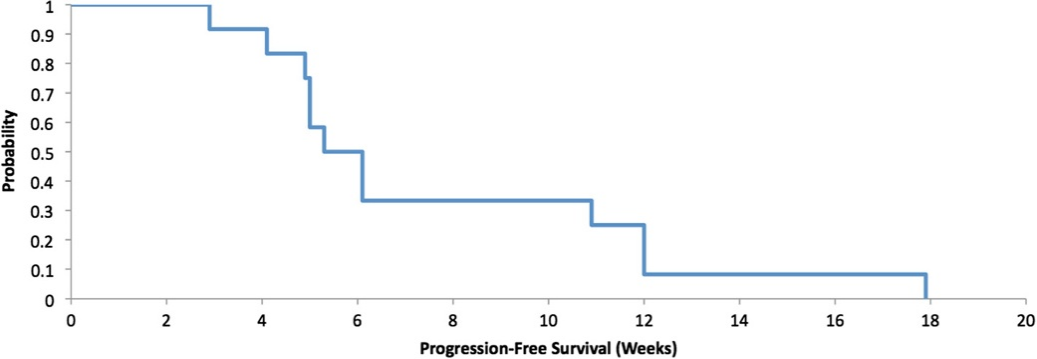
**Progression free survival.**

Table 3: Sixty-seven percent of the study subjects (N = 8) experienced Progressive Disease as their best response, and 33% of the study subjects (N = 4) experienced Stable Disease (Figure 1). Response was measured using RECIST criteria version 1.1. Table 3 illustrates summary data and shows that the duration of stable disease had a median value of 11.6 weeks (N = 4), with a minimum value of 10.9 weeks and a maximum value of 17.9 weeks. Progression free-survival had a median of 5.7 weeks (Figure 2). The 90% Confidence Interval for the response rate is {0 - 22%}.

**Table 3:** **Summary details**

Reason treatment ended:	
Progressive disease	N=12, 100%
Best response:	
Stable disease	N=4, 33.33%
Progressive disease	N=8, 66.67%
Number of treatment weeks completed	
N	12
Mean	7.9
Std	4.72
Min	4
Max	20
Duration of stable disease (weeks)	
N	4
Mean	13.03
Std	3.28
Min	10.9
Max	17.9
**Progression-free survival: median of 5.7 weeks.**	
Survival status	
Alive	N=1, 8.33%
Dead	N=11, 91.67%

## Discussion

In summary, 12 participants with metastatic Ewing sarcoma were enrolled, and all were evaluable. Two cases of grade 3 anemia, one case of grade 3 thrombocytopenia, one case of grade 3 decreased lymphocyte count, and one case of grade 4 other respiratory, thoracic and mediastinal disorder were observed. Otherwise, no significant or unexpected toxicities were observed while participants were receiving olaparib. There were 0 PR/CR, 4 SD (duration 10.9, 11.4, 11.9, and 17.9 wks), and 8 PD as best response. Of the participants who experienced SD, 2 had minor responses (−9.0% and −11.7% by RECIST 1.1, see Figure 1). Further enrollment was therefore discontinued after interim analysis.

This lack of clinical efficacy is in direct contrast to preclinical modeling that supported in vitro sensitivity of Ewing sarcoma cell lines to olaparib [9]. One explanation for this discrepancy is that some cell lines may have been derived from patient tumor samples that were not yet chemoresistant, while all participants in this study had proven relapse or progression after administration of standard chemotherapeutic agents for Ewing sarcoma. Several other factors that could explain the disparity include failure to achieve in vitro levels of olaparib at the clinical dose, secondary genomic or epigenomic alterations that might have activated other drivers of tumor cell proliferation that would render the PARP pathway nonessential, or yet unidentified mediators of PARP-rescue derived from tumor-environment interactions.

Preclinical work, however, does support potential activity of PARP inhibition when combined with alkylating agents. In fact, although Brenner et al. showed that in mice xenografts, Ewing sarcoma (RD-ES) cells treated with 100 mg/kg of olaparib twice a day still demonstrated tumor growth, albeit significantly slower than untreated controls [13], in that same report, olaparib combined with the alkylating agent, temozolomide, yielded exquisite and durable in vivo tumor response. Such trials combining PARP inhibition with chemotherapy are currently being conducted or under development at several sites across the U.S. and in Europe.

## Conclusion

This study is the first report of a prospective phase II trial to evaluate the safety and efficacy of a PARP inhibitor in patients with Ewing sarcoma. Olaparib tablets were well tolerated when administered to this small cohort at the 400 mg BID dose, although the median duration of dosing was for only 5.7 weeks. However, no significant responses were seen, and the short average interval to disease progression underscores the aggressiveness of this disease.
